# Divergent Roles of p75^NTR^ and Trk Receptors in BDNF's Effects on Dendritic Spine Density and Morphology

**DOI:** 10.1155/2012/578057

**Published:** 2012-03-27

**Authors:** Christopher A. Chapleau, Lucas Pozzo-Miller

**Affiliations:** Department of Neurobiology, SHEL-1002, Civitan International Research Center, The University of Alabama at Birmingham, 1825 University Boulevard, Birmingham, AL 35294-2182, USA

## Abstract

Activation of TrkB receptors by brain-derived neurotrophic factor (BDNF) followed by MAPK/ERK signaling increases dendritic spine density and the proportion of mature spines in hippocampal CA1 pyramidal neurons. Considering the opposing actions of p75^NTR^ and Trk receptors in several BDNF actions on CNS neurons, we tested whether these receptors also have divergent actions on dendritic spine density and morphology. A function-blocking anti-p75^NTR^ antibody (REX) did not affect spine density by itself but it prevented BDNF's effect on spine density. Intriguingly, REX by itself increased the proportion of immature spines and prevented BDNF's effect on spine morphology. In contrast, the Trk receptor inhibitor k-252a increased spine density by itself, and prevented BDNF from further increasing spine density. However, most of the spines in k-252a-treated slices were of the immature type. These effects of k-252a on spine density and morphology required neuronal activity because they were prevented by TTX. These divergent BDNF actions on spine density and morphology are reminiscent of opposing functional signaling by p75^NTR^ and Trk receptors and reveal an unexpected level of complexity in the consequences of BDNF signaling on dendritic morphology.

## 1. Introduction

The mammalian neurotrophins, a family of growth factors that include nerve growth factor (NGF), brain-derived neurotrophic factor (BDNF), neurotrophin-3 (NT-3), and neurotrophin 4/5 (NT-4/5), have essential roles in neuronal survival and differentiation [1, 2]. In addition to these classical functions, BDNF in particular has been shown to be one of the most potent modulators of synaptic transmission and plasticity, as well as neuronal and synaptic morphology [3–5]. Each neurotrophin exerts its actions through binding and activation of specific, membrane-bound tropomyosin-related kinase (Trk) receptors or a single pan-neurotrophin receptor, the so-called p75^NTR^ [6]. Individual Trk receptors have high affinity for specific neurotrophins: TrkA for NGF, TrkB for BDNF and NT-4, and TrkC for NT-3; on the other hand, all neurotrophins bind to p75^NTR^ with equal affinity and no apparent selectivity [7].

Neurotrophin binding to the aforementioned receptors, in addition to interactions between p75^NTR^ and Trk receptors, organizes complex signaling cascades that control various neuronal actions such as survival, differentiation, neurite and axonal outgrowth, and synaptic function during nervous system development [8–12]. Current work to examine neurotrophin receptors has added an intriguing level of complexity, specifically the opposing functional actions of p75^NTR^ and Trk receptors. Opposing receptor actions have been implicated in several neurotrophin functions, such as neuronal survival (Trk activates prosurvival signals, while p75^NTR^ leads to cell death), axonal outgrowth (Trk is a promoting signal, while p75^NTR^ inhibits axonal growth), and hippocampal synaptic plasticity (TrkB is necessary for long-term potentiation, LTP, while p75^NTR^ receptors are required for long-term depression, LTD) (reviewed by [13]). With respect to dendritic development, TrkB activation enhances dendritic growth [14, 15], while p75^NTR^ negatively regulates dendritic complexity in hippocampal neurons from adult mice [16].

Studies comparing the level of function of TrkB and p75^NTR^ during postnatal spinogenesis has not been extensively examined presumably because of the developmental deficits that exist in TrkB knockout mice [17]. Reports demonstrate that p75^NTR^ knockout mice display an increase in spine density and a significant reduction in the proportion of stubby spines in CA1 pyramidal neurons from hippocampal slice cultures [16]. While postnatal TrkB knockout mice (P13-14) demonstrate a reduction in synapse number in the hippocampus [18, 19], it should be noted that these results might be a consequence contributed to increased neuronal death also observed in this region [20]. Therefore, it remains to be determined if a functional antagonism exists between p75^NTR^ and Trk receptors in regards to BDNF-induced changes in spine density and form.

## 2. Material and Methods

### 2.1. Organotypic Slice Cultures

Hippocampal slice cultures were prepared from postnatal-day 7 to 10 (P7–P10) Sprague-Dawley rats and maintained *in vitro* as previously described [21, 22]. Briefly, rats were quickly decapitated and their brains aseptically dissected and immersed in ice-cold dissecting solution, consisting of Hanks' Balanced Salt Solution (HBSS), supplemented with glucose (36 mM) and antibiotics/antimycotics (1 : 100; penicillin/streptomycin/amphotericin B). Hippocampi were then dissected and transversely sectioned into ~500 *μ*m slices using a custom-made tissue slicer [23] strung with 20 *μ*m-thick tungsten wire (California Fine Wire Company; Grover Beach, CA). Slices were incubated at 4°C for ~30 min and then plated on tissue culture inserts (0.4 *μ*m pore size, Millicell-CM, Millipore Corporation; Billerica, MA). Culture media contained minimum essential media (MEM; 50%), HBSS (25%), heat-inactivated equine serum (20%), L-glutamine (1 mM), and D-glucose (36 mM). Slices were maintained in incubators set at 36°C, 5% CO_2_, and 98% relative humidity (Thermo-Forma, Waltham, MA). Culture medium was first changed at 4 days *in vitro* (div) and every 2 days afterwards.

### 2.2. Particle-Mediated Gene Transfer

After 7 days *in vitro*, slices were transfected as previously described [24]. Briefly, plasmid cDNA for enhanced yellow fluorescent protein (eYFP; Clontech; Mountain View, CA) was introduced by biolistic transfection using a Helios gene gun (Bio-Rad; Hercules, CA). Plasmid cDNA was precipitated onto 1.6 *μ*m-diameter colloidal gold at a ratio of 2 *μ*g DNA/1 mg gold and then coated onto Tefzel tubing. Slices on tissue culture inserts were bombarded with gold particles accelerated by ~100 psi He from a distance of 2 cm using a modified gene gun nozzle. Prior to transfection, an antibiotic/antimycotic mixture (1 : 100; penicillin/streptomycin/amphotericin B) was added to culture media to prevent contamination during biolistic transfection. The antibiotic/antimycotic mixture was only used during biolistic transfection and was removed after 24 hrs to avoid the consequences of network desinhibition from their known actions on GABA_A_ receptor channels [25].

### 2.3. Treatment Conditions

Slices were kept in a serum containing media throughout the course of the experiments and were randomly assigned to the following groups: (1) control, serum-containing media; (2) BDNF (250 ng/mL); (3) anti-p75^NTR^ antibody REX (50 *μ*g/mL), known to block p75^NTR^ function [26] (provided by L. Reichardt, UCSF); (4) BDNF in the presence of REX antibody; (5) k-252a (200 nM, in DMSO; Calbiochem; San Diego, CA) to block autophosphorylation and activation of tyrosine kinase domains of plasma membrane neurotrophin receptors [27]; (6) BDNF in the presence of k-252a; (7) TTX (1 *μ*M; Alomone Labs; Jerusalem, Israel); (8) TTX in the presence of k-252a. The DMSO concentration never exceeded 0.01%, which did not affect any of the parameters under study. In experiments where BDNF was added in the presence of k-252a or REX, these compounds were added 30 min before application of BDNF. A droplet (50 *μ*L) of medium was gently applied onto each slice to facilitate penetration, followed by full medium exchange (1 mL per tissue culture well). In each slice culture preparation (which came from the same litter of P7 rat pups), slices were randomly assigned to 1 of the 8 experimental groups (including controls). The 8 different experimental treatments (including controls) were applied to at least 2 different culture plates from at least 2 different culture preparations from 2 different litters of P7 rat pups (sometimes weeks apart). Each culture preparation had its own control group, and we have at least 3 different sets of control cultures coming from 3 different culture preparations from 3 different litters of P7 rat pups. All treatments lasted 48 hrs, beginning 48 hrs after biolistic transfection, and slices were coded for subsequent blind quantitative analyses of dendritic spine density and morphology by an investigator unaware of treatment groups.

### 2.4. Laser Scanning Confocal Microscopy

After 48 hrs in each of the treatment conditions, slices were fixed by immersion in 4% paraformaldehyde in 100 mM phosphate buffer (overnight at 4°C) and washed in phosphate buffer saline (PBS). Filter membranes around each slice were trimmed, and each slice was individually mounted on glass slides and coverslipped using Vectashield (Vector Laboratories; Burlingame, CA). Transfected pyramidal neurons located in the CA1 region displaying eYFP fluorescence throughout the entire dendritic tree and lacking signs of degeneration (e.g., dendritic blebbing) were selected for confocal imaging. High-resolution images of secondary and tertiary branches of apical dendrites were acquired with a Fluoview FV-300 laser scanning confocal microscope (Olympus; Center Valley, PA) using an oil immersion 100x (NA 1.4) objective lens (PlanApo). eYFP was excited using the 488 nm line of an Argon laser and detected using standard FITC filters. Series of optical sections in the *z*-axis were acquired at 0.1 *μ*m intervals through each dendritic branch.

### 2.5. Analysis of Spine Density

Dendritic spines of CA1 pyramidal neurons were identified as small protrusions that extended ≤3 *μ*m from the parent dendrite and counted offline in maximum-intensity projections of the *z*-stacks using ImageJ software (National Institutes of Health), as described [28]. Protrusions longer than 3 *μ*m were rarely observed in CA1 pyramidal neurons in slice cultures at this developmental age (P7–P10 harvesting, 11 days *in vitro*) and since likely represent dendritic filopodia, they were not considered in the following analyses. Care was taken to ensure that each spine was counted only once by following its projection course through the stack of *z*-sections. Spines were counted only if they appeared continuous with the parent dendrite. Spine density was calculated by quantifying the number of spines per dendritic segment and normalized to 10 *μ*m of dendrite length. Microscope calibrations were performed using 1.07 *μ*m fluorescent microspheres (Polysciences Inc.; Warrington, PA), which yielded a lateral resolution of 10.8 pixels per *μ*m (i.e., 92 nm per pixel).

### 2.6. Measurements of Spine Dimensions for Spine-Type Classification

**Figure 1:**
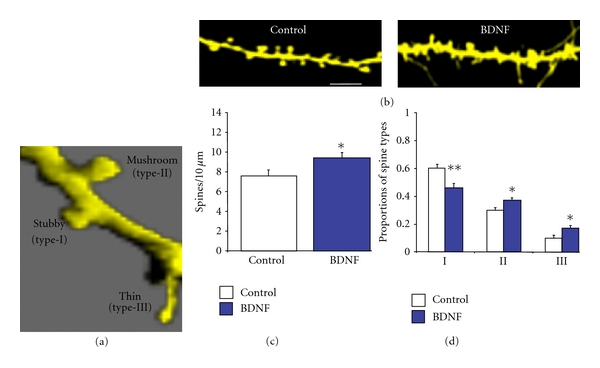
BDNF increases dendritic spine density and affects the proportion of morphological spine types. (a) Dendritic segment of a CA1 pyramidal neuron that was volume-rendered to illustrate individual spine geometrical dimensions and examples of different spine types. (b) Representative examples of dendritic segments of CA1 pyramidal neurons maintained in serum-containing media (SM) and treated with BDNF (250 ng/mL) for 48 hrs (scale bar represents 2 *μ*m). (c) Dendritic spine density expressed per 10 *μ*m of apical dendrite. (d) Proportion of each morphological type of dendritic spine, expressed as a fraction of the total spine population. *P < 0.05 and **P < 0.01, after an unpaired Student's *t*-test.

The categorization of different morphological spine types was performed as described [29]. Briefly, spines were classified into three classical subjective categories [30, 31], but based on objective geometric measurements of their dimensions. Spines were classified as stubby (type I), mushroom (type II), or thin (type III) types based on the *L*/*N* and *H*/*N* ratios, where *L* is spine length, *H* is the maximum head width, and *N* is the maximum neck width [32, 33]. Following these criteria, stubby spines have a length that is similar to the diameter of the neck and is similar to the diameter of the head (*L* ≈ *N* ≈ *H*), mushroom spines have a greater *H*/*N* ratio (*H* > *N*), and the length of thin spines is much greater than their neck diameters (*L* ≫ *N*) (Figure  1(a)). The majority (~65%) of these dendritic spines have presynaptic partners as assessed by synaptobrevin staining [28], and despite morphological differences, all the three spine types make synaptic contacts *in vitro* [34]. Spine dimensions were measured in maximum-intensity projections of the *z*-stacks using ImageJ by an investigator unaware of treatment groups.

### 2.7. Statistical Analyses

Data were analyzed statistically using unpaired Student's *t*-test or analysis of variance (ANOVA) followed by Tukey's procedure for multiple comparisons using Prism (GraphPad; San Diego, CA). *P* < 0.05 was considered significant. Data are presented as mean ± standard error of the mean (SEM).

## 3. Results

**Figure 2:**
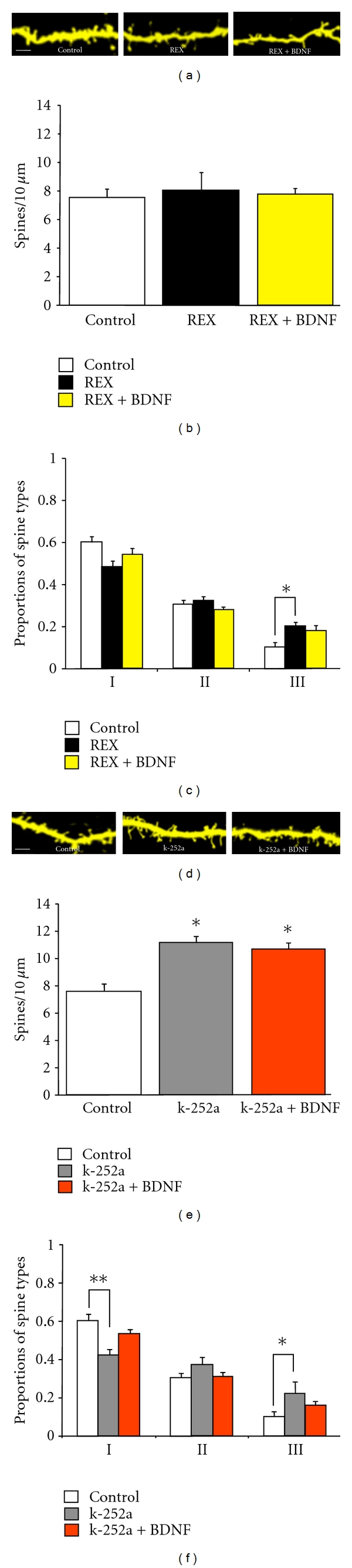
Role of Trk and p75^NTR^ in BDNF's effects on dendritic spine density and morphology. (a) Representative examples of dendritic segments of CA1 pyramidal neurons maintained in serum-containing media (SM) and treated with the function-blocking antibody of p75^NTR^, REX (50 *μ*g/mL), and BDNF (250 ng/mL) for 48 hrs (scale bar represents 2 *μ*m). (b) Dendritic spine density expressed per 10 *μ*m of apical dendrite. (c) Proportion of each morphological type of dendritic spine, expressed as a fraction of the total spine population. (d) Representative examples of dendritic segments of CA1 pyramidal neurons maintained in SM and treated with k-252a (200 nM) and BDNF (250 ng/mL) for 48 hrs. (e) Dendritic spine density expressed per 10 *μ*m of apical dendrite. (f) Proportion of each morphological type of dendritic spines, expressed as a fraction of the total spine population. *P < 0.05, **P < 0.01, and ***P < 0.001, after a one-way ANOVA.

**Figure 3:**
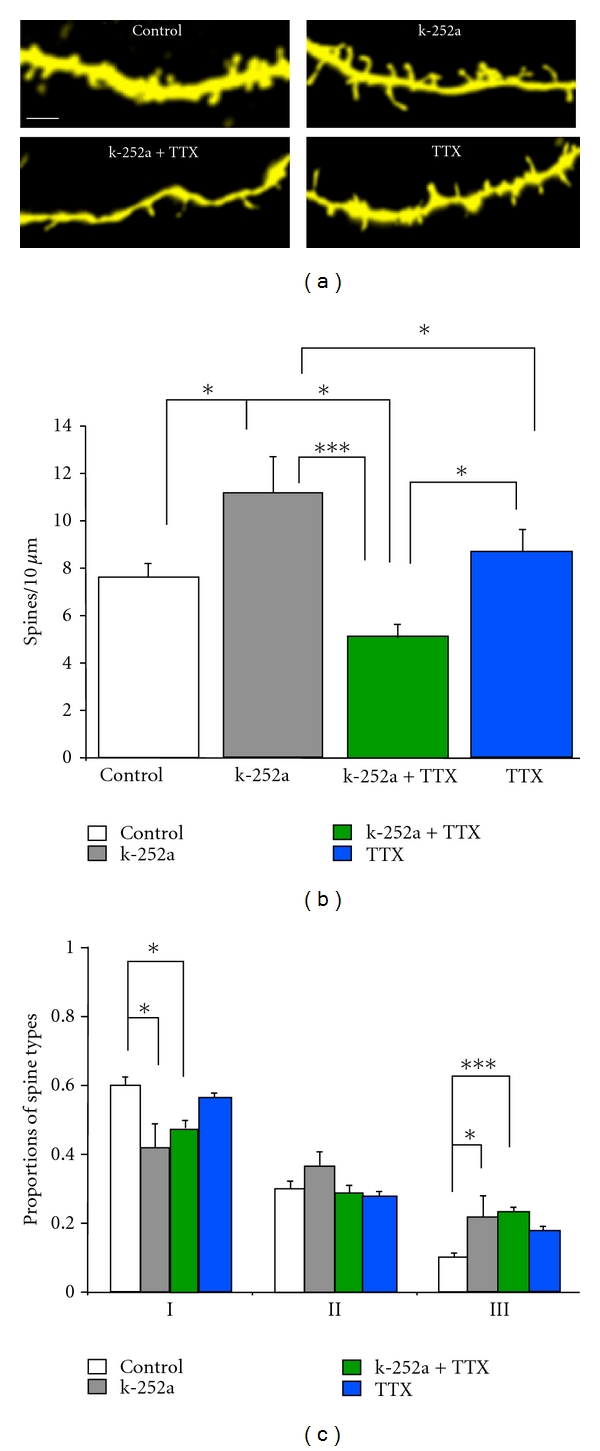
Role of neuronal activity in k-252a's effects on dendritic spine density and morphology. (a) Representative examples of dendritic segments of CA1 pyramidal neurons maintained in serum-containing media (SM) and treated with k-252a (200 nM), TTX (1 *μ*M), or both k-252a and TTX for 48 hrs (scale bar represents 2 *μ*m). (b) Dendritic spine density expressed per 10 *μ*m of apical dendrite. (c) Proportion of each morphological type of dendritic spines, expressed as a fraction of the total spine population. *P < 0.05, **P < 0.01, and ***P < 0.001, after a one-way ANOVA.

Organotypic cultures from P7–10 rat hippocampal slices were biolistically transfected with eYFP and fixed 96 hrs after transfection. Confocal images of secondary and tertiary apical dendrites of CA1 pyramidal neurons were collected (Figures 1(b), 2(a), 2(d), and 3(a)), and the density and dimensions of individual dendritic spines were measured as previously described [21].  Table 1 has the results of the quantitative analyses of spine density and morphology, and Table 2 has the number of slices, neurons, and spines counted and measured in each treatment group, as well as the total dendritic length analyzed. Because serum removal for 48 hrs reduced the expression of TrkB and p75^NTR^ receptors in cultured slices [21], and BDNF (250 ng/mL) increased spine density in the presence of serum (Control = 7.60 ± 0.57 spines/10 *μ*m versus BDNF = 9.39 ± 0.56 spines/10 *μ*m, 10 cells from 7 slices; *P* < 0.05; Figure  1(c)) [21], for the current studies we used serum-containing media. In addition, BDNF affected spine morphology by decreasing the proportion of stubby spines (Control = 0.60 ± 0.03, 13 cells/9 slices versus BDNF = 0.46 ± 0.01, 10 cells/7 slices; *P* < 0.01) and increasing the proportion of mushroom spines (SM = 0.30 ± 0.02 versus SM + BDNF = 0.37 ± 0.01; *P* < 0.05), as well as thin spines (SM = 0.10 ± 0.02 versus SM + BDNF = 0.17 ± 0.02; *P* < 0.05; Figure  1(d)) [21].

## 4. Role of p75^NTR^ on BDNF's Actions on Dendritic Spine Density and Morphology

To test the role of p75^NTR^ in BDNF's effects on dendritic spines, we used the function-blocking anti-p75^NTR^ antibody REX [26] at a concentration (50 *μ*g/mL) that blocked p75^NTR^-dependent LTD induction in acute hippocampal slices [35]. Blocking p75^NTR^ function for 48 hrs had no effect on spine density by itself (REX: 8.04 ± 1.32 spines/10 *μ*m, 6 cells/3 slices versus Control: 7.60 ± 0.57 spines/10 *μ*m, 13 cells/9 slices; *P* = 0.907; Figure 2(b)). However, BDNF failed to increase dendritic spine density in the presence of REX (REX + BDNF: 7.82 ± 0.39 spines/10 *μ*m, 10 cells/4 slices versus REX or Control; *P* = 0.907; Figure 2(b)).

Intriguingly, REX increased the proportion of thin spines (thin type III in REX: 0.20 ± 0.02, 6 cells/3 slices versus Control: 0.10 ± 0.02; 13 cells/9 slices; *P* < 0.05), without affecting the proportion of the other spine types (stubby type I in REX: 0.48 ± 0.03 versus Control 0.60 ± 0.03; *P* = 0.07) (mushroom type II in REX: 0.32 ± 0.02 versus Control: 0.30 ± 0.02; *P* = 0.444) (Figure 2(c)). This effect is reminiscent to the reduction in the proportion of stubby spines in p75^NTR^ knockout mice [16]. Furthermore, BDNF failed to change the proportion of spine types in the presence of the anti-p75^NTR^ REX antibody (thin type III in REX + BDNF: 0.18 ± 0.02; 10 cells/4 slices; *P* > 0.05 versus Control) (stubby type I in REX + BDNF: 0.54 ± 0.03; *P* = 0.07 versus Control) (mushroom type II in REX + BDNF: 0.28 ± 0.01; *P* = 0.444 versus Control; Figure 2(c)). Taken altogether, these results demonstrate that BDNF requires functional p75^NTR^ to increase dendritic spine density and modulate dendritic spine morphology.

### 4.1. Role of Trk Receptors on BDNF's Actions on Dendritic Spine Density and Morphology

We next blocked the kinase activity of Trk receptors with k-252a [27]. We previously reported that k-252a (200 nM) applied for 5–9 days *in vitro* led to a significant reduction in spine density in CA1 pyramidal neurons from hippocampal slice cultures maintained in serum-free media [28]. Surprisingly, exposure to k-252a for a shorter time (48 hs) and in the presence of horse serum in the media significantly increased spine density in CA1 pyramidal neurons (k-252a: 11.2 ± 1.51 spines/10 *μ*m, 5 cells/5 slices; *P* < 0.05 versus Control; Figure 2(e)). In contrast to its blockade of BDNF's effects in serum-free slices [28], k-252a failed to prevent the effects of BDNF to increase spine density (k-252a + BDNF: 10.7 ± 0.8 spines/10 *μ*m, 15 cells/12 slices; *P* < 0.05 versus Control). However, BDNF did not further increase spine density in the presence of k-252a (*P* > 0.05 versus k-252a alone).

The increase in spine density by k-252a was unexpected; however, the majority of these spines were of the thin immature type. Indeed, k-252a increased the fraction of thin type III spines (k-252a: 0.22 ± 0.06, 5 cells/5 slices; *P* < 0.05 versus Control) and decreased the proportion of stubby type I spines (k-252a: 0.42 ± 0.07; *P* < 0.01 versus Control; Figure 2(f)). In addition, k-252a prevented BDNF to change the proportion of morphological spine types (k-252a + BDNF type I: 0.53 ± 0.02; type II: 0.31 ± 0.01; type III: 0.16 ± 0.01; 15 cells/12 slices; all *P* > 0.05 versus Control).

Considering the unexpected increase in dendritic spine density induced by the Trk inhibitor k-252a—albeit mostly of the long and thin type III category—and the role of neuronal activity in spine number and form [36], we tested whether the effect of k-252a required neuronal activity in the form of Na^+^-dependent action potentials. Indeed, TTX (1 *μ*M) prevented the effect of k-252a (200 nM) (TTX + k-252a: 5.05 ± 0.51 spines/10 *μ*m, 14 cells/7 slices; *P* < 0.001 versus k-252a; Figure 3(b)). Furthermore, exposure to both TTX and k-252a caused a loss of dendritic spines compared to slices maintained in the control serum media conditions (TTX + k-252a: 5.05 ± 0.51 spines/10 *μ*m, 14 cells/7 slices versus Control: 7.60 ± 0.57 spines/10 *μ*m, 13 cells/9 slices; *P* < 0.05; Figure 3(b)). It should be noted that this short exposure to TTX (48 hs) did not affect spine density (TTX: 8.60 ± 1.03 spines/10 *μ*m, 7 cells/5 slices; *P* > 0.05 versus Control; Figure 3(b)). On the other hand, TTX did not prevent the morphological spine changes induced by k-252a, including the increase in the proportion of thin type III spines induced by k-252a (TTX + k-252a: 0.23 ± 0.02; 14 cells/7 slices *P* < 0.001 versus Control; Figure 3(c)) and the reduction of stubby type I spines (TTX + k-252a: 0.47 ± 0.03; 14 cells/7 slices *P* < 0.05 versus Control; Figure 3(c)). The apparent increase in the proportion of thin spines in the TTX group did not reach statistical significance (TTX: 0.17 ± 0.02, 7 cells/5 slices; *P* > 0.05 versus Control; Figure 3(c)). These results suggest that ongoing BDNF signaling through TrkB receptors and spontaneous neuronal activity are intimately related in dendritic spine maintenance, as well as in the structural maturation of those morphological spine types thought to represent the postsynaptic compartment of mature synapses [37].

## 5. Discussion

To address the role of each BDNF receptor on dendritic spine density and morphology, we blocked either Trk or p75^NTR^ for 48 hs in the absence or presence of BDNF. We observed that brief exposures to the Trk inhibitor k-252a caused a significant increase in spine density in CA1 pyramidal neurons. However, most of these spines were of the thin category, thought to be highly motile and unstable structures characteristic of immature synapses [38–40]. The fact that longer exposures to k-252a by itself caused spine loss [28] suggests that an initial increase in thin immature spines precedes spine pruning [36]. In contrast, p75^NTR^ blockade with the function-blocking antibody REX prevented BDNF's effect on spine density. The importance of BDNF-induced modifications on neuron structure and physiology are well documented and continue to emerge. Since BDNF binds and activates two different receptors, determining how each receptor influences dendritic remodeling will provide greater understanding into the function of BDNF in synaptic plasticity. The observations reported here reflect a functional antagonism between p75^NTR^ and TrkB receptor signaling in the maintenance of dendritic spines.

Consistent with our previous study [7], BDNF increased spine density and shifted the proportion of spine types towards the thin and mushroom-shaped spines in hippocampal slice cultures maintained in serum-containing media. We also uncovered that antagonism of either p75^NTR^ or Trk receptors increased the proportion of thin (type-III) spines. It has been suggested that thin spines represent “learning spines” due to their highly motile and unstable nature, while mushroom spines are “memory spines” because they are highly stable [41]. Since inhibition of either BDNF receptor increased the proportion of thin spines, we speculate that BDNF participates in the formation/maintenance/pruning of these “learning spines.” However, the difference between these two receptor systems is in their ability to differentially modulate spine density. Blocking Trk signaling with the tyrosine kinase inhibitor k-252a, caused a significant increase in spine density, while blocking p75^NTR^ with the function-blocking antibody REX had no effect on spine density. These results strongly suggest the existence of a sustained tone of BDNF signaling that contributes to dendritic spine maintenance in a manner dependent on the activation of Trk receptors (but not p75^NTR^). Taken together, these results suggest that during postnatal development, p75^NTR^ activation is important for initial dendritic spine formation, while Trk receptors participate in dendritic spine maintenance at later developmental stages. Indeed, conditional deletion of TrkB in postnatal forebrain excitatory neurons caused a reduction in spine density and a higher proportion of long and thin spines in hippocampal and primary visual cortex neurons [42–44], suggesting that sequential activation of TrkB receptors followed by p75^NTR^ might be critical for BDNF-mediated modulation of dendritic spine density and morphology. Evidence of such developmental differences has been observed in subventricular zone-derived neurons, where p75^NTR^ activation modulates dendritic growth in early stages of development, while TrkB activation plays a role in later stages [45].

Ongoing neuronal activity was required for the unexpected effect of the Trk inhibitor k-252a : TTX prevented the increase in spine density and proportion of thin immature spines induced by k-252a. Intriguingly, there was a dramatic loss of spines in slice cultures exposed to both TTX and k-252a compared to control serum media conditions. While the specific mechanisms of dendritic spine maintenance and pruning remain somewhat unknown, it is well accepted that ongoing levels of synaptic transmission and the ensuing intracellular Ca^2+^ levels contribute in a significant manner [36]. Consistent with this view, silencing neuronal activity for 7 days *in vitro* with TTX reduced spine density in CA1 pyramidal neurons in slice cultures [46]. The spine loss in those week-long silencing experiments likely results from prolonged absence of excitatory synaptic input [47]. It should be noted that a shorter period of neuronal inactivity (TTX for 2 days *in vitro*) did not cause spine loss, but rather altered the proportion of morphological spine types favoring the thin and immature spine type [29]. Our present results suggest that ongoing Trk signaling is required for spine maintenance in TTX-silenced slice cultures, revealing a novel aspect of activity-dependent maintenance and pruning of dendritic spines in hippocampal pyramidal neurons.

The observations on dendritic spine density and morphology reported here may reflect the functional antagonism between p75^NTR^ and Trk receptor signaling [13]. Even though current reports indicate that p75^NTR^ and Trk do not directly interact, it has been proposed that these receptor complexes share similar downstream signaling pathways to create more complex actions [48]. On the other hand, p75^NTR^ can act as a coreceptor for Trk receptors, creating high affinity sites for Trk receptor activation [49]. Furthermore, the interaction of truncated TrkB receptors (TrkB. T1) with p75^NTR^ enhanced dendritic filopodia outgrowth in the absence of neurotrophin binding [50]. Thus, signaling through the two BDNF receptors may have different consequences for dendritic spine density and morphology depending on whether they are activated alone, in concert or under different levels of ongoing neuronal activity. Taken altogether, these studies have revealed an unexpected level of complexity in the consequences of BDNF signaling on dendritic morphology.

## Figures and Tables

**Table 1:** Quantitative results of dendritic spine analyses.

	Spine density (per 10 *μ*m)	Spine length (*μ*m)	Head width (*μ*m)	Neck width (*μ*m)	Proportion of type I (stubby)	Proportion of type II (mushroom)	Proportion of type III (thin)
Serum media (SM)	7.60 ± 0.57	0.57 ± 0.02	0.37 ± 0.01	0.30 ± 0.01	0.60 ± 0.03	0.30 ± 0.02	0.10 ± 0.02
SM + BDNF	9.39 ± 0.56	0.62 ± 0.01	0.37 ± 0.01	0.26 ± 0.01	0.46 ± 0.01	0.37 ± 0.01	0.17 ± 0.02
SM + REX	8.04 ± 1.32	0.67 ± 0.04	0.39 ± 0.02	0.29 ± 0.02	0.48 ± 0.03	0.32 ± 0.02	0.20 ± 0.02
SM + REX + BDNF	7.82 ± 0.39	0.61 ± 0.03	0.37 ± 0.01	0.28 ± 0.01	0.54 ± 0.03	0.28 ± 0.01	0.18 ± 0.02
SM + k-252a	11.16 ± 1.51	0.65 ± 0.07	0.34 ± 0.01	0.24 ± 0.01	0.42 ± 0.07	0.37 ± 0.04	0.22 ± 0.06
SM + k-252a + BDNF	10.72 ± 0.77	0.63 ± 0.02	0.37 ± 0.01	0.29 ± 0.01	0.53 ± 0.02	0.31 ± 0.01	0.16 ± 0.01
SM + TTX + k-252a	5.05 ± 0.51	0.71 ± 0.02	0.37 ± 0.02	0.28 ± 0.01	0.47 ± 0.03	0.29 ± 0.02	0.23 ± 0.02
SM + TTX	8.60 ± 1.03	0.59 ± 0.02	0.34 ± 0.01	0.27 ± 0.01	0.56 ± 0.02	0.27 ± 0.02	0.17 ± 0.02

**Table 2:** Summary of total dendritic length, cells, slices, and individual spines sampled for the quantitative dendritic spine analyses.

Condition	Total dendritic length (*μ*m)	Number of cells	Number of slices	Total spines counted and measured
Serum Media (SM)	1,706.34	13	9	1,309
SM + BDNF	2,131.13	10	7	2,056
SM + REX	1,482.14	6	3	1,238
SM + REX + BDNF	2,513.30	10	4	2,021
SM + k-252a	1,196.25	5	5	1,468
SM + k-252a + BDNF	5,221.77	15	12	5,965
SM + TTX + k-252a	2,225.84	14	7	1,077
SM + TTX	1,431.73	7	5	1,307
